# Molecular Characterizations of Kenyan Brachiaria Grass Ecotypes with Microsatellite (SSR) Markers

**DOI:** 10.3390/agronomy7010008

**Published:** 2017-02-09

**Authors:** Naftali Ondabu, Solomon Maina, Wilson Kimani, Donald Njarui, Appolinaire Djikeng, Sita Ghimire

**Affiliations:** 1 Biosciences Eastern and Central Africa-International Livestock Research Institute (BecA-ILRI) Hub, P.O. Box 30709, Nairobi 00100, Kenya; gondabu@yahoo.com (N.O.); solomon.dna.maina@gmail.com (S.M.); wilkims2005@yahoo.com (W.K.); A.Djikeng@cgiar.org (A.D.); 2 Kenya Agricultural and Livestock Research Organization (KALRO), P.O. Box 57811, Nairobi 00200, Kenya; donaldnjarui@yahoo.com (D.N.)

**Keywords:** analysis of molecular variance, breeding, fixation index, genetic conservation, private allele

## Abstract

Brachiaria grass is an emerging forage option for livestock production in Kenya. Kenya lies within the center of diversity for *Brachiaria* species, thus a high genetic variation in natural populations of *Brachiaria* is expected. Overgrazing and clearing of natural vegetation for crop production and nonagricultural uses and climate change continue to threaten the natural biodiversity. In this study, we collected 79 *Brachiaria* ecotypes from different parts of Kenya and examined them for genetic variations and their relatedness with 8 commercial varieties. A total of 120 different alleles were detected by 22 markers in the 79 ecotypes. Markers were highly informative in differentiating ecotypes with average diversity and polymorphic information content of 0.623 and 0.583, respectively. Five subpopulations: International Livestock Research Institute (ILRI), Kitui, Kisii, Alupe, and Kiminini differed in sample size, number of alleles, number of private alleles, diversity index, and percentage polymorphic loci. The contribution of within-the-individual difference to total genetic variation of Kenyan ecotype population was 81%, and the fixation index (*F*_ST_ = 0.021) and number of migrant per generation (*N*m = 11.58) showed low genetic differentiation among the populations. The genetic distance was highest between Alupe and Kisii populations (0.510) and the lowest between ILRI and Kiminini populations (0.307). The unweighted neighbor-joining (NJ) tree showed test ecotypes grouped into three major clusters: ILRI ecotypes were present in all clusters; Kisii and Alupe ecotypes and improved varieties grouped in clusters I and II; and ecotypes from Kitui and Kiminini grouped in cluster I. This study confirms higher genetic diversity in Kenyan ecotypes than eight commercial varieties (Basilisk, Humidicola, Llanero, Marandú, MG4, Mulato II, Piatá and Xaraés) that represent three species and one three-way cross-hybrid Mulato II. There is a need for further collection of local ecotypes and their morphological, agronomical, and genetic characterizations to support Brachiaria grass breeding and conservation programs.

## 1. Introduction

Brachiaria grass is one of the most important tropical grasses distributed throughout the tropics, especially in Africa [1]. The genus *Brachiaria* consists of about 100 documented species of which 7 perennial species of African origin have been used for pasture production in South America, Asia, South Pacific, and Australia [2]. It has high biomass production potential and produces nutritious herbage resulting in increased livestock productivity [3,4]. Brachiaria is adapted to drought and low-fertility soils, sequesters carbon through its large root system, enhances nitrogen use efficiency, and subsequently minimizes eutrophication and greenhouse gas emissions [5–8]. *Brachiaria* plays important roles in soil erosion control and ecological restoration. *Brachiaria* species have been an important component of sown pastures in humid lowlands and savannas of tropical America, with current estimated acreage of 99 million hectares in Brazil alone [9].

In Africa, the evaluations of *Brachiaria* species for pasture improvement started during the 1950s. These researches focused on *B. brizantha*, *B. decumbens*, *B. mutica*, and *B. ruziziensis* for forage production, agronomy (establishment, drought, cutting intervals, and fertilizers), compatibility with herbaceous and tree legumes, nutritive values, and their benefit to ruminant production. These studies concluded the suitability and broader adaptation of several *Brachiaria* species to different agroecological zones in Africa [10]. However, these practices were not widespread because of ample communal grazing lands, limited realization on roles of sown pasture in the livestock production, subsistence animal farming, and low government priority to pasture development. Recently, the mounting demand for livestock products in Africa has renewed interest of farmers, researchers, development agencies, and government organizations on forages, particularly in species with good adaptability to climate change such as Brachiaria grass. Therefore, there has been multiple repatriations of Brachiaria grass to Africa in the form of hybrids and improved landraces [11,12]. These materials have shown positive performance in terms of biomass production, improved forage availability and livestock productivity in Kenya and Rwanda. These results have revealed *Brachiaria* as an ideal forage option for the livestock farmers in East Africa.

Despite high popularity, the Brachiaria acreage in Africa is low and relies on a few varieties that were developed for tropical Americas and Australia. Within a short period of introduction, some of these varieties have shown susceptibility to pests and diseases, raising question on the expansion of Brachiaria acreage in Africa with these varieties. There is therefore a need for an Africa-based *Brachiaria* improvement program to develop varieties that are tolerant to biotic and abiotic stress for different environmental conditions. Germplasms of broad genetic base is the prerequisite for any crop improvement. The best approach to increase genetic variations in apomictic species such as *Brachiaria* is tapping natural variations from the center of diversity. Since the 1950s, multiple missions were undertaken in Africa to collect *Brachiaria* germplasms, with a current inventory of 987 accessions of 33 known *Brachiaria* species [13]. Considering distribution of *Brachiaria* in Africa and size of the continent, the number of samples available in collection is definitely non-exhaustive and warrants further collection efforts. However, the existence of these genetic resources in Africa is continuously threatened by overgrazing and clearing of vegetation for crop production and nonagricultural uses and adverse effects of climate change.

Kenya is located within a region that represents a center of diversity for genus *Brachiaria*. Therefore, high natural variation is expected among *Brachiaria* populations in Kenya. This study aimed to create a collection of local *Brachiaria* ecotypes in Kenya, assess their genetic diversity using microsatellite markers, and examine their genetic relationships with eight commercial cultivars. The study will broaden geographical coverage and/or genetic base of the global *Brachiaria* collection and provide invaluable information for *Brachiaria* improvement and conservation programs.

## 2. Results

### 2.1. Descriptive Statistics for Simple Sequence Repeat (SSR) Markers

Descriptive statistics for all marker sets were computed (Table 1). The major allele frequency ranged from 0.2405 (Brz3002) to 0.8228 (Brz0076) with a mean of 0.5184. The number of different alleles ranged from 3 (Brz0029) to 10 (Brz0130) with a mean of 5.45. The genetic diversity averaged to 0.6225 with a range of 0.3169–0.8021. Similarly, the polymorphic information content (PIC) ranged from 0.3087 (Brz0076) to 0.8384 (Brz3002) with a mean of 0.5825.

**Table 1:** Descriptive statistics for microsatellite markers.

Marker	MAF	*N*_DA_	*I*	*PIC*
Brz0012	0.4304	5	0.7101	0.6670
Brz0028	0.4304	5	0.6521	0.5892
Brz0029	0.6203	3	0.5124	0.4327
Brz0067	0.4051	5	0.7419	0.7061
Brz0076	0.8228	3	0.3169	0.3087
Brz0087	0.481	8	0.6983	0.6649
Brz0092	0.8101	5	0.3352	0.3240
Brz0100	0.4684	4	0.6614	0.6052
Brz0115	0.3671	7	0.8021	0.7829
Brz0117	0.6076	6	0.5371	0.4676
Brz0118	0.5063	4	0.5573	0.4613
Brz0122	0.4557	6	0.6739	0.6225
Brz0130	0.3418	10	0.7947	0.7706
Brz0149	0.7722	5	0.3874	0.3679
Brz0156	0.6456	4	0.5365	0.497
Brz0203	0.3671	7	0.7685	0.7379
Brz0212	0.5823	8	0.6195	0.5906
Brz0213	0.7468	4	0.4192	0.3932
Brz0214	0.4304	7	0.7432	0.7138
Brz0235	0.4051	4	0.7438	0.709
Brz3002	0.2405	5	0.854	0.8384
Brz3009	0.4684	5	0.6313	0.5643
Mean	0.5184	5.45	0.6225	0.5825

MAF = minor allele frequency, *N*_DA_ = number of different alleles, *I* = Shannon’s genetic diversity, and *PIC* = polymorphic information content.

### 2.2. Population Diversity Indices

The population diversity indices for five ecotype populations from Kenya were summarized (Table 2). The International Livestock Research Institute (ILRI) population had highest number of different alleles, and the Alupe population had the least. The number of private alleles was highest for the ILRI population and the lowest for Kisii population. The information index ranged from 0.408 to 0.887 with a mean of 0.599. The observed heterozygosity was higher than expected for all populations. The percentage polymorphic loci ranged from 46.47% (Kitui) to 86.87% (ILRI).

**Table 2:** Summary of population diversity indices averaged over 22 simple sequence repeat (SSR) markers.

Population	*N*	*N*a	*N*p	*A*e	*I*	*H*o	*H*e	*PL* (%)
ILRI	60	3.633	0.833	2.21	0.887	0.76	0.499	86.67
KITUI	3	1.233	0.133	1.171	0.408	0.417	0.261	46.67
KISII	5	1.567	0.067	1.396	0.498	0.537	0.315	56.67
ALUPE	4	1.6	0.0133	1.486	0.524	0.544	0.333	60.00
KIMIN	7	2.133	0.1	1.833	0.678	0.647	0.41	70.00
Mean	15.8	2.033	0.22926	1.619	0.599	0.581	0.364	64.00

*N* = number of samples, *N*a = number of different Alleles, *N*p = number of private alleles, *A*e = number of effective alleles, *I* = Shannon’s information Index, *H*o = observed heterozygosity, *H*e = expected heterozygosity and *PL* = percentage polymorphic loci.

### 2.3. Genetic Diversity and Relationships

**Figure 1:**
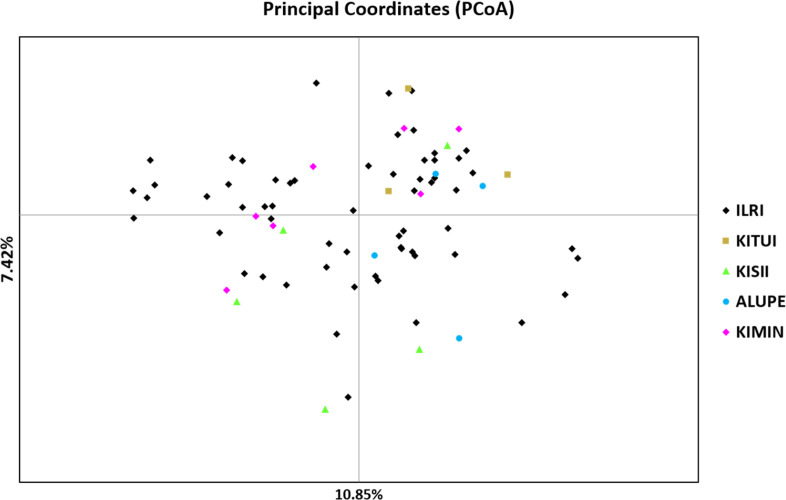
Principal coordinates analysis (PCoA) biplot showing the clustering of the 79 *Brachiaria* ecotypes from different parts of Kenya (orange = Kitui, black = International Livestock Research Institute (ILRI) Farm, green = Kisii, blue = Alupe, and purple = Kiminini).

**Figure 2:**
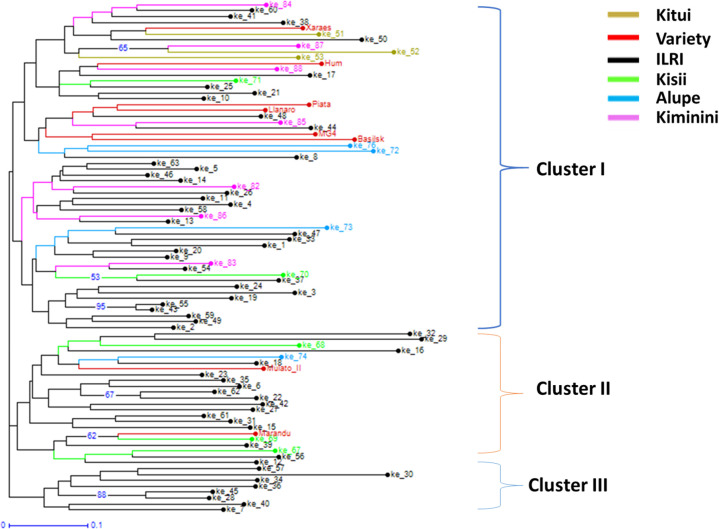
Unweighted neighbor-joining tree using the simple matching dissimilarity coefficient based on 22 microsatellite loci for all collected 79 *Brachiaria* ecotypes (ke_1 to ke_88) collected from different parts of Kenya (orange = Kitui, red = commercial cultivars, black = ILRI Farm, green = Kisii, blue = Alupe, and purple = Kiminini), and 8 commercial cultivars (*B. brizantha* cvs. Marandú, MG4, Piatá, and Xaraés; *B. decumbens* cv. Basilisk; *B. humidicola* cvs. Humidicola and Llanero; and three-species-ways cross-hybrid Mulato II.

The pairwise genetic distance and population matrix of Nie genetic identity were calculated (Table 3). The genetic distance was highest between Alupe and Kitui populations (0.510), whereas it was the lowest between ILRI and Kiminini populations (0.307). Similarly, genetic identity was the highest between ILRI and Kiminini populations (0.636) and the lowest between Alupe and Kitui populations (0.235). The principal coordinate analysis (PCoA) plot of ecotypes from five populations showed no distinct clustering pattern (Figure 1). The first two principal coordinates explained 18.27% of the total genetic variation within the test ecotypes. Specifically, the first and second coordinates explained 10.85% and 7.42% of the total genetic variation, respectively. However, an unweighted neighbor-joining tree of 79 ecotypes and 8 commercial cultivars showed them grouped into three distinct clusters (Figure 2). Cluster I included 50 ecotypes from all five populations and six cultivars, cluster II included 21 ecotypes from three populations (Alupe, ILRI, and Kisii) and two cultivars, and cluster III included 8 ecotypes, all from the ILRI population.

**Table 3:** Pairwise genetic distance based on shared allele (left) and population matrix of Nie genetic identity (right) among the *Brachiaria* ecotype population from Kenya.

Population	Alupe	ILRI	Kiminini	Kisii	Kitui
Alupe	-	0.462	0.388	0.323	0.235
ILRI	0.393	-	0.636	0.440	0.327
Kiminini	0.448	0.307	-	0.399	0.299
Kisii	0.467	0.392	0.446	-	0.247
Kitui	0.510	0.441	0.413	0.503	-

### 2.4. Analysis of Molecular Variance (AMOVA)

The partitioning of the total variation in population at different levels was estimated with AMOVA (Table 4). Within-the-individual difference contributed highest (81%) to total variation followed by among-individual difference (17%) and among-population differences (2%). The fixation index (*F*_ST_) and number of immigration per generation (*N*m) for study populations were 0.021 and 11.585, respectively.

**Table 4:** Analysis of molecular variance among and within populations, and within individuals for *Brachiaria* accessions based on 22 SSR loci.

Source	Degree of Freedom	Sum of Squares	Mean Squares	Estimated Variance	Variation (%)	*p* Values
Among Populations	4	43.440	10.860	0.155	2%	0.023
Among Individual	74	619.649	8.374	1.215	17%	0.001
Within Individual	79	469.500	5.943	5.943	81%	0.001
Total	157	1132.589		7.313	100%	
*F*_ST_ = 0.021 and *N*m = 11.580						

*F*_ST_ = Fixation index; *N*m = Number of migration per generation.

## 3. Discussion

The genetic complexity, primarily apomictic mode of reproduction, and abundant natural variations in Africa urge for a two-pronged approach (selection and breeding) for improving Brachiaria grass in Africa. All-inclusive germplasm base with documented variations are prerequisite for the effective breeding programs. This study collected 79 *Brachiaria* ecotypes in Kenya and documented their genetic variations using microsatellite markers.

The PIC values for 22 SSR markers averaged to 0.5825, suggesting markers were capable of differentiating 79 Kenyan *Brachiaria* ecotypes. The PIC value in this study is within the range reported by Silva et al. [14], Jungmann et al. [15], and Vigan et al. [16], but was lower than that found by Jungmann et al. [17] and Pessoa-Filho et al. [18]. Similarly, the average numbers of allele detected per loci (5.45) was in the range reported by Silva et al. [14], Jungmann et al. [15], and Vigan et al. [16], but was about half and one-third of that reported by Jungmann et al. [17] and Pessoa-Filho et al. [18], respectively. However, these comparisons between studies may not be conclusive due to differences in types and number of germplasms and markers used among studies.

The analysis of the distributions of alleles across populations is important for explaining genetic diversity and population relationships [19]. Private alleles are important in plant breeding and conservation as they are present only in a single population among a broader collection of populations [20]. Five ecotypes populations of Kenya were different for private alleles, with the highest number of private alleles in the ILRI population and the least in the Kiminini population. Such variations in the private alleles among populations most likely was the effect of the number of individuals per population, which ranged from 3 to 60 individuals. Although no information was available on species composition of each population, it is likely the presence of multiple species resulting in a high number of private alleles in some populations. Irrespective of populations, *H*_O_ was higher than *H*_E_, indicating mixing of previously isolated populations. This is consistent with the human involvement in moving planting materials and outcrossing nature of some *Brachiaria* species, for example, *B. ruziziensis*.

The study population varied in genetic distance and genetic identity coefficients. The highest genetic distance between Alupe and Kitui populations can be explained by the wider geographical distance between these two locations (675 km), but the genetic distance between other populations could not be associated to geographical proximity. Reports are available on forage research, including seed production of *B. ruziziensis* in Kitale, Kenya [21,22], and involvement of Kenya Agricultural Research Institute and Kenya Seed Company in the past in production and trading of *B. ruziziensis* seeds [23]. It is likely that some of these *Brachiaria* seeds might have reached farmers’ fields and other research stations in Kenya, including the ILRI, and afterwards naturalized in the wild. If this hold true, a low genetic distance (0.307) between the ILRI and Kiminini (20 km away from Kitale) populations could be because of shared genetic materials in early days.

The contribution of within-individual difference to total variation was 81%, whereas among-theindividual and among-populations differences contributed 17% and 2%, respectively (Table 5). These observations were in agreement with Vigna et al. [16] and Pessoa-Filho et al. [18], who reported high contributions of within-the-accession/individual differences to total variation in *B. brizantha* (84%) and *B. ruziz*iensis (88%) populations. Similarly, Garcia et al. [24] and Azevedo et al. [25] reported 73% and 65% of total variation attributed to within species or cluster, respectively. However, Jungmann et al. [26] reported 44% of the variation in *B. humidicola* accessions as being due to the subdivision of the germplasms into five groups. The *F*_ST_ and effective number of migrants per generation (*N*m) values of 0.021 and 11.580 indicated a relatively low genetic differentiation among populations [27] and relatively high level of gene flow among the Kenyan ecotypes populations [28], respectively. A low genetic differentiation among the study populations could be associated with apomictic mode of reproduction, variable ploidy causing meiotic anomalies leading to reduced pollen fertility, and dispersal of seeds by migratory herbivorous and human activities such as hay transportation for feeding animals [16,26,29–32]. Polyploid plants are effective colonizers that can occupy pioneer habitats and generate individuals that are able to exploit new niches or outcompete progenitor species, whereas apomictic polyploid plants can fix heterosis [16,26,30].

**Table 5:** Microsatellite markers, primer sequences, annealing temperature (*T*a), allele sizes, and number of repeat motifs (adapted from Silva et al. [14]).

Marker	Forward Primer	Reverse Primer	*Ta*(_C)	Allele Size (bp)	Repeat Motif
Brz0012	ACTCAAACAATCTCCAACACG	CCCACAAATGGTGAATGTAAC	59	160	(AT)^8^
Brz0028	CATGGACAAGGAGAAGATTGA	TGGGAGTTAACATTAGTGTTTTT	57	158	(TA)^8^
Brz0029	TTTGTGCCAAAGTCCAAATAG	TATTCCAGCTTCTTCTGCCTA	56	150	(AG)^14^
Brz0067	TTAGATTCCTCAGGACATTGG	TCCTATATGCCGTCGTACTCA	51	156	(AT)^9^
Brz0076	CCTAGAATGCGGAAGTAGTGA	TTACGTGTTCCTCGACTCAAC	58	151	(AT)^7^
Brz0087	TTCCCCCACTACTCATCTCA	AACAGCACACCGTAGCAAGT	60	243	(GA)^9^
Brz0092	TTGATCAGTGGGAGGTAGGA	TGAAACTTGTCCCTTTTTCG	54	251	(AT)^6^
Brz0100	CCATCTGCAATTATTCAGGAAA	GTTCTTGGTGCTTGACCATT	56	256	(AT)^11^
Brz0115	AATTCATGATCGGAGCACAT	TGAACAATGGCTTTGAATGA	59	252	(AT)^6^
Brz0117	AGCTAAGGGGCTACTGTTGG	CGCGATCTCCAAAATGTAAT	60	260	(TA)^5^
Brz0118	AGGAGGTCCAAATCACCAAT	CGTCAGCAATTCGTACCAC	57	252	(CT)^11^
Brz0122	CATTGCTCCTCTCGCACTAT	CTGCAGTTAGCAGGTTGGTT	57	253	(CA)^6^
Brz0130	TCCTTTCATGAACCCCTGTA	CATCGCACGCTTATATGACA	57	248	(CT)^14^
Brz0149	GCAAGACCGCTGTTAGAGAA	CTAACATGGACACCGCTCTT	57	245	(AT)^11^
Brz0156	GCCATGATGTTTCATTGGTT	TTTTGCACCTTTCATTGCTT	58	260	(AC)^7^
Brz0203	CGCTTGAGAAGCTAGCAAGT	TAGCCTTTTGCATGGGTTAG	57	301	(GA)^8^
Brz0212	ACTCATTTTCACACGCACAA	CGAAGAATTGCAGCAGAAGT	57	301	(CA)^5^
Brz0213	TGAAGCCCTTTCTAAATGATG	GAACTAGGAAGCCATGGACA	57	296	(CA)^7^
Brz0214	TCTGGTGTCTCTTTGCTCCT	TCCATGGTACCTGAATGACA	57	309	(AT)^8^
Brz0235	CACACTCACACACGGAGAGA	CATCCAGAGCCTGATGAAGT	57	298	(TC)^9^
Brz3002	GCTGGAATCAGAATCGATGA	GAACTGCAGTGGCTGATCTT	57	160	(AAT)^7^
Brz3009	AGACTCTGTGCGGGAAATTA	ACTTCGCTTGTCCTACTTGG	55	151	(AAT)^10^

This study is an effort to build a collection of *Brachiaria* ecotypes in Kenya and identify the potential values of these genetic resources in the *Brachiaria* breeding program. It is important to acknowledge some methodological limitations of this study while inferring population genetic parameters such as unequal and/or small sample sizes (3–60 individuals per population), unknown species and ploidy status of ecotypes, and dominant scoring scheme used in recording SSR fragments. The current *Brachiaria* taxonomy is far from satisfactory and the problem of generic identity, and species composition across entire taxa needs to be undertaken [1,22]. Application of robust genetic markers and bioinformatics procedures in genetic analysis of *Brachiaria* spp. have been constrained by a limited understanding of *Brachiaria* genetics, cytogenetic and reproductive biology, and unavailability of reference genome. The agricultural and environmental importance of *Brachiaria* has recently spurred several studies on *Brachiaria*, including sequencing of *B. ruziziensi*s genome. Therefore, Kenyan ecotypes collected in this study should be conserved and characterized further with the advent of new genomics and bioinformatics tools developed for species with complex genome.

This is among the very first studies of this century in sub-Saharan Africa that involved collection of local *Brachiaria* ecotypes from different parts of Kenya and examination of their genetic differences using microsatellite markers. The genetic diversity data revealed that ecotypes, though representing a few locations of Kenya, contained much more diversity than currently available 8 improved *Brachiaria* varieties, which represent three species (*B. brizantha*, *B. decumbens*, and *B. humidicola*) and three-way cross-hybrid Mulato II (*B. brizantha* × *B. decumbens* × *B. ruziziensis*). These results clearly indicate a need for (I) further collection of local ecotypes in Kenya and other east and central African countries that represent center of diversity of *Brachiaria* species to enrich the *Brachiaria* genepool in the gene bank collections; (II) genetic characterization of local ecotypes and currently available gene bank materials to understand diversity and ascertain the need for further collection; and (III) morphological characterization of available genetic resource to identify/develop varieties suitable for different production environments.

## 4. Experimental Section

### 4.1. Source of Plant Materials

Whole plant sample of 79 *Brachiaria* ecotypes were collected from five different parts of Kenya: Alupe (*n* = 4), ILRI Farm (*n* = 60), Kiminini (*n* = 7) Kisii (*n* = 5), and Kitui (*n* = 3) in 2013 and 2014, and maintained in field at forage research plots of International Livestock Research Institute (ILRI), Headquarters, Nairobi, Kenya. Seeds of eight improved varieties—*B. decumbens* cv. Basilisk, *B. brizantha* cvs. Marandú, Xaraés, Piatá, and MG4, *B. humidicola* cvs. Humidicola and Llanero (Marangatu Sementes, Ribeirão Preto, São Paulo, Brazil), and Mulato II (Tropical Seeds, Coral Springs, FL, USA)—were planted in a variety demonstration plot at the ILRI Campus. About 4-week-old leaves were harvested from all 79 ecotypes and 8 varieties (one sample/variety), freeze-dried, and stored at −80^◦^ prior to DNA extraction. Ecotypes from all location but the ILRI Campus were collected jointly by Biosciences eastern and central and Africa-International Livestock Research Institute (BecA-ILRI) Hub and Kenya Agricultural and Livestock Research Organization (KALRO). The collection details are summarized in Table 6.

**Table 6:** Collection details of Kenyan *Brachiaria* ecotypes included in the diversity assessment.

Ecotype	Species	Status	Location	Alt. (m a.s.l.)	Lat. (S)	Lon. (E)	Collection Year
ke_1	*Brachiaria* spp.	Wild	ILRI Farm	1761	1.27085	36.72204	2013
ke_2	*Brachiaria* spp.	Wild	ILRI Farm	1783	1.27091	36.72200	2013
ke_3	*Brachiaria* spp.	Wild	ILRI Farm	1787	1.27117	36.72206	2013
ke_4	*Brachiaria* spp.	Wild	ILRI Farm	1805	1.27152	36.72212	2013
ke_5	*Brachiaria* spp.	Wild	ILRI Farm	1798	1.27306	36.72255	2013
ke_6	*Brachiaria* spp.	Wild	ILRI Farm	1804	1.27307	36.72384	2013
ke_7	*Brachiaria* spp.	Wild	ILRI Farm	1810	1.27292	36.72390	2013
ke_8	*Brachiaria* spp.	Wild	ILRI Farm	1813	1.27281	36.72404	2013
ke_9	*Brachiaria* spp.	Wild	ILRI Farm	1815	1.27269	36.72436	2013
ke_10	*Brachiaria* spp.	Wild	ILRI Farm	1814	1.27262	36.72483	2013
ke_11	*Brachiaria* spp.	Wild	ILRI Farm	1808	1.27275	36.72517	2013
ke_12	*Brachiaria* spp.	Wild	ILRI Farm	1871	1.27077	36.72224	2013
ke_13	*Brachiaria* spp.	Wild	ILRI Farm	1814	1.27076	36.72532	2013
ke_14	*Brachiaria* spp.	Wild	ILRI Farm	1870	1.27073	36.72562	2013
ke_15	*Brachiaria* spp.	Wild	ILRI Farm	1852	1.27088	36.72697	2013
ke_16	*Brachiaria* spp.	Wild	ILRI Farm	1851	1.27091	36.72702	2013
ke_17	*Brachiaria* spp.	Wild	ILRI Farm	1840	1.27135	36.72716	2013
ke_18	*Brachiaria* spp.	Wild	ILRI Farm	1836	1.27152	36.72699	2013
ke_19	*Brachiaria* spp.	Wild	ILRI Farm	1832	1.27214	36.72649	2013
ke_20	*Brachiaria* spp.	Wild	ILRI Farm	1830	1.27236	36.72605	2013
ke_21	*Brachiaria* spp.	Wild	ILRI Farm	1828	1.2725	36.72592	2013
ke_22	*Brachiaria* spp.	Wild	ILRI Farm	1823	1.27268	36.72547	2013
ke_23	*Brachiaria* spp.	Wild	ILRI Farm	1825	1.27263	36.72520	2013
ke_24	*Brachiaria* spp.	Wild	ILRI Farm	1825	1.27273	36.72519	2013
ke_25	*Brachiaria* spp.	Wild	ILRI Farm	1825	1.27261	36.72560	2013
ke_26	*Brachiaria* spp.	Wild	ILRI Farm	1833	1.27213	36.72660	2013
ke_27	*Brachiaria* spp.	Wild	ILRI Farm	1835	1.27196	36.72673	2013
ke_28	*Brachiaria* spp.	Wild	ILRI Farm	1843	1.27144	36.72709	2013
ke_29	*Brachiaria* spp.	Wild	ILRI Farm	1852	1.27109	36.72713	2013
ke_30	*Brachiaria* spp.	Wild	ILRI Farm	1876	1.27067	36.72585	2013
ke_31	*Brachiaria* spp.	Wild	ILRI Farm	1837	1.27086	36.72210	2014
ke_32	*Brachiaria* spp.	Wild	ILRI Farm	1882	1.27084	36.72208	2014
ke_33	*Brachiaria* spp.	Wild	ILRI Farm	1854	1.27252	36.72235	2014
ke_34	*Brachiaria* spp.	Wild	ILRI Farm	1839	1.27264	36.72424	2014
ke_35	*Brachiaria* spp.	Wild	ILRI Farm	1826	1.27274	36.72518	2014
ke_36	*Brachiaria* spp.	Wild	ILRI Farm	1824	1.27233	36.72612	2014
ke_37	*Brachiaria* spp.	Wild	ILRI Farm	1830	1.27257	36.72567	2014
ke_38	*Brachiaria* spp.	Wild	ILRI Farm	1835	1.27165	36.72692	2014
ke_39	*Brachiaria* spp.	Wild	ILRI Farm	1847	1.27101	36.72718	2014
ke_40	*Brachiaria* spp.	Wild	ILRI Farm	1871	1.27077	36.72536	2014
ke_41	*Brachiaria* spp.	Wild	ILRI Farm	1866	1.2708	36.72210	2014
ke_42	*Brachiaria* spp.	Wild	ILRI Farm	1859	1.27134	36.72213	2014
ke_43	*Brachiaria* spp.	Wild	ILRI Farm	1842	1.27285	36.72249	2014
ke_44	*Brachiaria* spp.	Wild	ILRI Farm	1835	1.27242	36.72230	2014
ke_45	*Brachiaria* spp.	Wild	ILRI Farm	1829	1.2734	36.72302	2014
ke_46	*Brachiaria* spp.	Wild	ILRI Farm	1828	1.27315	36.72381	2014
ke_47	*Brachiaria* spp.	Wild	ILRI Farm	1829	1.27271	36.72427	2014
ke_48	*Brachiaria* spp.	Wild	ILRI Farm	1828	1.27269	36.72454	2014
ke_49	*Brachiaria* spp.	Wild	ILRI Farm	1816	1.27261	36.72550	2014
ke_50	*Brachiaria* spp.	Wild	ILRI Farm	1829	1.2717	36.72688	2014
ke_51	*Brachiaria* spp.	Wild	Kitui	1163	NA	NA	2014
ke_52	*Brachiaria* spp.	Wild	Kitui	1163	NA	NA	2014
ke_53	*Brachiaria* spp.	Wild	Kitui	1163	NA	NA	2014
ke_54	*Brachiaria* spp.	Wild	ILRI Farm	1754	1.27778	36.38821	2014
ke_55	*Brachiaria* spp.	Wild	ILRI Farm	1857	1.2708	36.72206	2014
ke_56	*Brachiaria* spp.	Wild	ILRI Farm	1856	1.27284	36.72204	2014
ke_57	*Brachiaria* spp.	Wild	ILRI Farm	1844	1.27162	36.72208	2014
ke_58	*Brachiaria* spp.	Wild	ILRI Farm	1840	1.27203	36.72217	2014
ke_59	*Brachiaria* spp.	Wild	ILRI Farm	1822	1.2732	36.72357	2014
ke_60	*Brachiaria* spp.	Wild	ILRI Farm	1822	1.27321	36.72358	2014
ke_61	*Brachiaria* spp.	Wild	ILRI Farm	1810	1.27281	36.72506	2014
ke_62	*Brachiaria* spp.	Wild	ILRI Farm	1821	1.27176	36.72678	2014
ke_63	*Brachiaria* spp.	Wild	ILRI Farm	1824	1.27155	36.72697	2014
ke_67	*Brachiaria* spp.	Wild	Kisii	1750	0.68575	34.78978	2014
ke_68	*Brachiaria* spp.	Wild	Kisii	1750	0.68486	34.78914	2014
ke_69	*Brachiaria* spp.	Wild	Kisii	1750	0.68484	34.78910	2014
ke_70	*Brachiaria* spp.	Wild	Kisii	1750	0.68471	34.78896	2014
ke_71	*Brachiaria* spp.	Wild	Kisii	1750	0.68473	34.78884	2014
ke_72	*Brachiaria* spp.	Wild	Alupe	1200	0.49766	34.12480	2014
ke_73	*Brachiaria* spp.	Wild	Alupe	1200	0.49781	34.12480	2014
ke_74	*Brachiaria* spp.	Wild	Alupe	1200	0.49847	34.12319	2014
ke_76	*Brachiaria* spp.	Wild	Alupe	1200	0.49855	34.12284	2014
ke_82	*Brachiaria* spp.	Wild	Kiminini	1750	0.89104	34.91368	2014
ke_83	*Brachiaria* spp.	Wild	Kiminini	1750	0.89102	34.91378	2014
ke_84	*Brachiaria* spp.	Wild	Kiminini	1750	0.89126	34.91338	2014
ke_85	*Brachiaria* spp.	Wild	Kiminini	1750	0.89144	34.91310	2014
ke_86	*Brachiaria* spp.	Wild	Kiminini	1750	0.89139	34.91302	2014
ke_87	*Brachiaria* spp.	Wild	Kiminini	1750	0.8913	34.91272	2014
ke_88	*Brachiaria* spp.	Wild	Kiminini	1750	0.89131	34.91264	2014

### 4.2. Genomic DNA Extraction

The DNA was extracted using the cetyl-trimethyl ammonium bromide (CTAB) [33] method with slight modifications. About 150 mg of the young leaves were cut into small pieces, ground in liquid nitrogen, and added with 800 µL of 2% CTAB buffer. The suspension was transferred into clean microfuge tubes and incubated at 65 ^◦^C for 30 min, followed by incubation at room temperature for 5 min and centrifuged at 3500 rpm for 10 min. After centrifugation, 400 µL of supernatant was transferred into new microfuge tubes and 400 µL of chloroform iso-amyl alcohol (24:1) was added to each tube and mixed by inversion for 10 min. Tubes were spun at 3500 rpm for 10 min, aqueous phase was transferred to clean microfuge tubes, and 400 µL of chloroform iso-amyl alcohol (24:1) was added again to each tube and spun for 10 min at 1100 rpm; this process was repeated twice. After the final centrifugation, the DNA was precipitated in 300 µL of cold isopropanol (100%) and inverted about 50 times to facilitate the mixing and precipitation, and incubated overnight at −20 ^◦^C. The following day, the microfuge tubes were removed from the freezer, thawed and spun at 3500 rpm at 4 ^◦^C for 20 min. The isopropanol was decanted and the genomic DNA pellet was air-dried. The DNA pellet was rinsed with 300 µL of 70% (*w*/*v*) ethanol and dissolved in 100 µL of low-salt TE buffer containing 3 µL of 10 mg/mL of 1% RNase solution and incubated in a water bath at 45 ^◦^C for 90 min. DNA quality and quantity were checked by 0.8% agarose gel (*w*/*v*) and NanoDrop Spectrophotometer. The genomic DNA was adjusted to the final concentration of 20 ng/µL and stored at 4 ^◦^C for PCR amplification.

### 4.3. PCR Amplification and Genotyping

The genomic DNA was amplified using AccuPower^®^PCRPreMix with Bioneer negative dye (Bioneer, Alameda, CA, USA). A reaction volume of 10 µL containing 0.4 µL MgCl_2_ (final concentration of 2 mM MgCl_2_), 0.4 µL each of forward and reverse primers labeled with different fluorescent dyes (6-FAM (blue), VIC (green), NED (black), and PET(red)), 2 µL template DNA (20 ng/µL), and 6.8 µL of sterile distilled water was used for PCR amplification. A total of 22 SSR markers (Table 5) initially developed for *B. ruziziensis* with the proven transferability to other species were used in this study [14]. The PCR conditions were: initial denaturation for 5 min at 94 ^◦^C followed by 35 cycles at 94 ^◦^C for 30 s, 57 ^◦^C for 60 s, 72 ^◦^C for 2 min, and final extension at 72 ^◦^C for 10 min. The amplicons’ integrity was checked using agarose gel electrophoresis in 2% agarose gel (*w*/*v*) stained with 2.5 µL of GelRed solution. The agarose gel images were visualized under Ultra-Violet and the digital image was captured. The size of amplified fragments was estimated comparing with 1 kb DNA ladder (Thermo Fisher Scientific, Waltham, MA, USA). The SSR fragment sizes and allele variations in the repeats were assessed by capillary electrophoresis of amplicons and sequencing of the amplified loci. The multiplexed PCR products were mixed with 8.87 µL Hi-Di-formamide and 0.135 µL fluorescent-labeled GeneScan™ LIZ size standard (Applied Biosystems, Foster City, CA, USA) in a 96-well microtiter plate. The mixed products were denatured at 95 ^◦^C for 3 min and snap-chilled on ice for 5 min to avoid the formation of double-strand DNA. The products were loaded to Applied Biosystems 3730xl DNA Analyzer (Applied Biosystems, Foster City, CA, USA).

### 4.4. Data Analysis

The allele sizes generated by all 22 SSR markers on 79 ecotypes and 8 commercial varieties were scored using GeneMapper v4.1 software (Applied Biosystems, Foster City, CA, USA). Since the information on ploidy levels of test ecotypes was not available, SSR fragments were analyzed following a dominant scoring scheme, as used for other polyploidy species [34–37]. ALS-Binary and Allelobin software [38,39] were used to convert allelic data to binary data (0, 1) where 0 and 1 represent absence and presence of an allele, respectively. Statistical analysis of allelic and binary data was performed using PowerMarker v.3.25 [40] to obtain total number of alleles per locus, allele size range, genetic diversity and heterozygosity, and frequency-based genetic distances were calculated using shared alleles distance matrix. The population diversity indices (e.g., number of alleles, private alleles, and effective alleles per locus, Shannon Information index, and observed and expected heterozygosity) were calculated using GenAIEx v.6.5 [41]. The same software was used to compute analysis of molecular variance (AMOVA), principal coordinate analysis (PCoA), and matrix of genetic distance. The Dice binary similarity coefficient [42] was used to generate the unweighted neighbor-joining tree (NJT) showing relationships among test genotypes in Darwin Software v6.0 [43].

## 5. Conclusions

Brachiaria is a native African grass which is widely distributed in Kenya. It is one of the most extensively cultivated forages in tropical Americas, Australia and East Asia. However the cultivation of Brachiaria for pasture production in Kenya and Africa in general has been recently initiated through the repatriation of Brachiaria in the form of hybrids and improved landraces from South America. Despite excellent herbage production performance and benefits to livestock productivity, some of these introduced materials have shown susceptibility to pests and diseases within a short period of establishment. It has raised serious concern on the expansion of Brachiaria acreage in Kenya urging the needs for the Africa based *Brachiaria* improvement program. This study with collection of 79 Brachiaria ecotypes from a few locations of Kenya and their genetic diversity analyses revealed the presence of substantial genetic variations among Kenyan ecotypes, and close genetic relationships among improved landraces and Hybrid Mulato II. This study suggests need for collecting more ecotypes from different agroecological regions of Kenya to broaden genetic bases of existing genebank collections, and their morphological, agronomical, and genetic characterizations to support Brachiaria grass breeding and conservation programs.
